# Supplementary figures and images for: Activated heme synthesis regulates glycolysis and oxidative metabolism in breast and ovarian cancer cells
Source: PLoS One. 2021 Nov 22;16(11):e0260400. doi: 10.1371/journal.pone.0260400 (PMC8608300; doi:10.1371/journal.pone.0260400)

Fig. 3

A

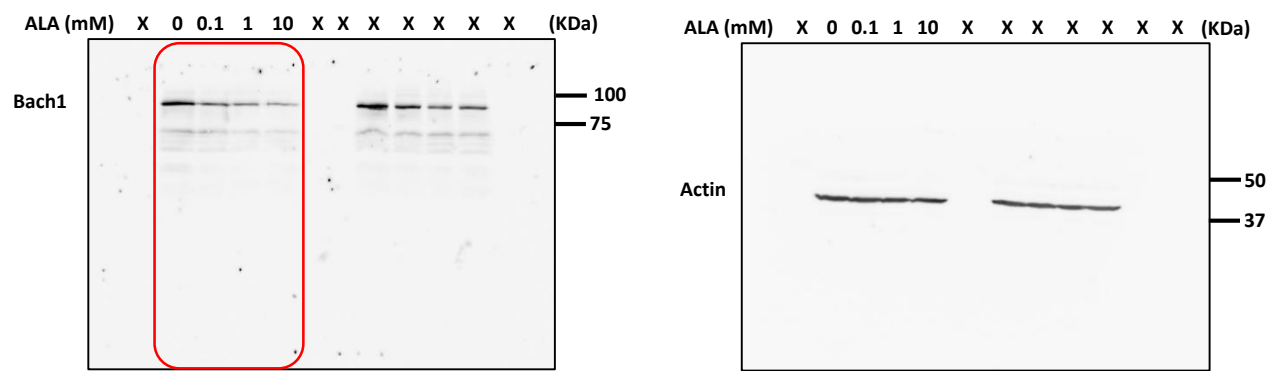

B

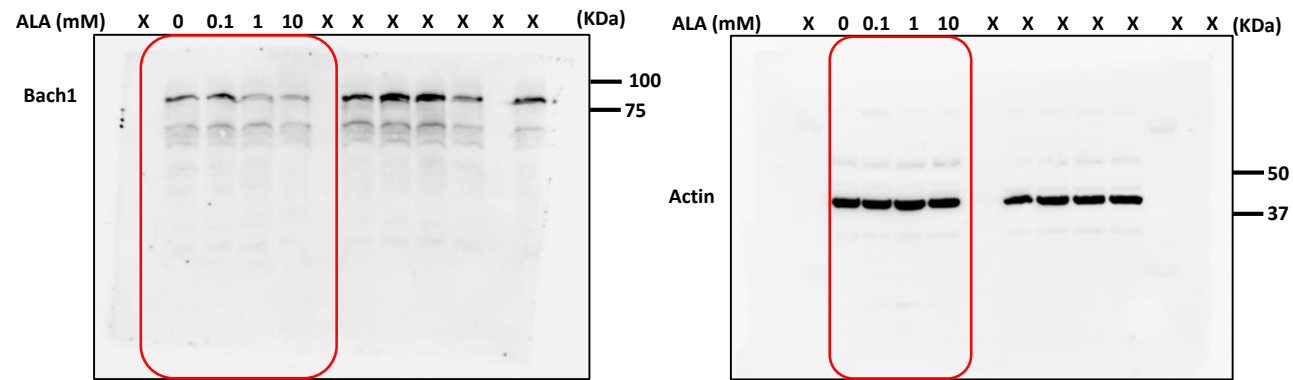

Fig. 3  
C

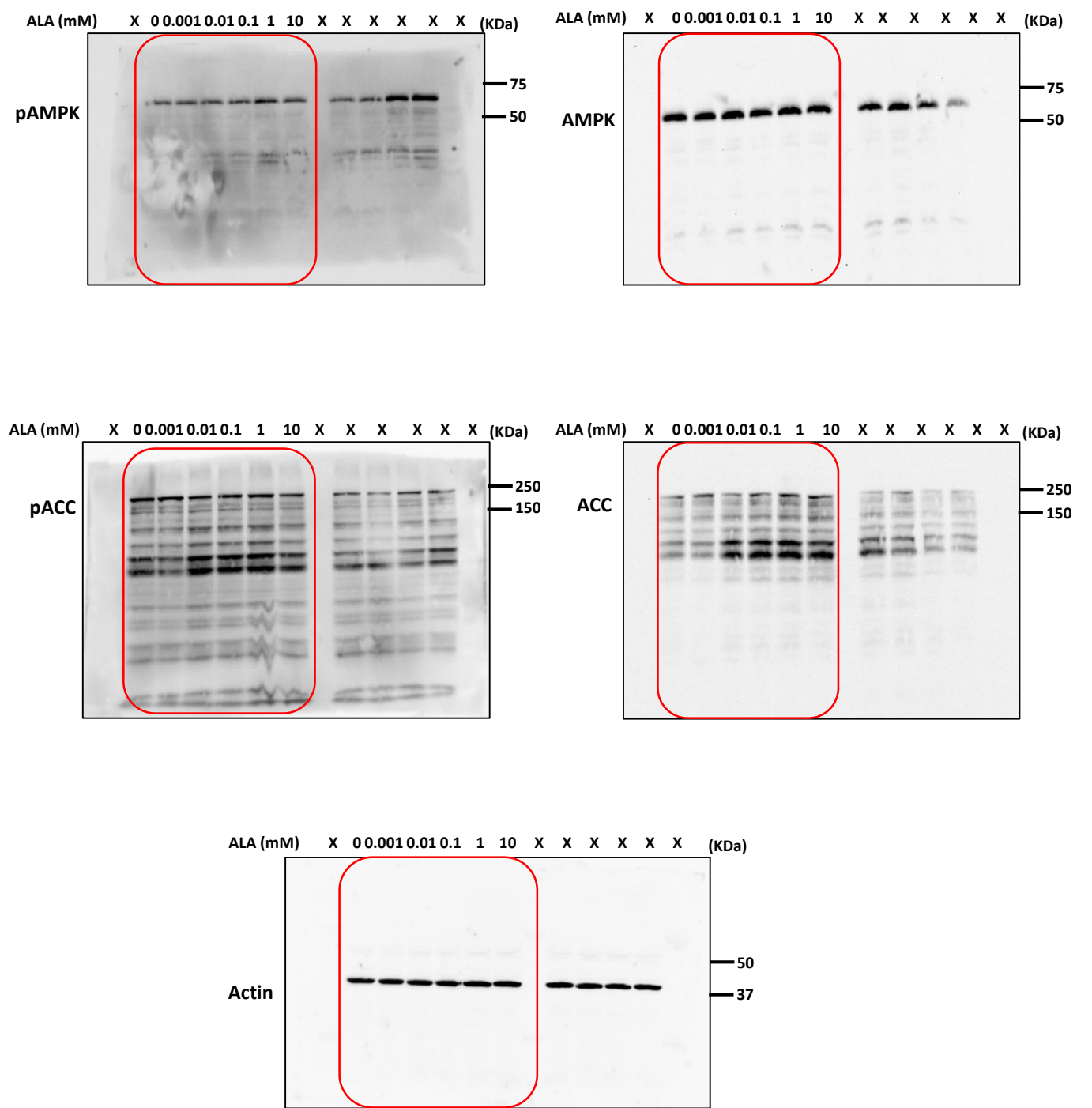

D

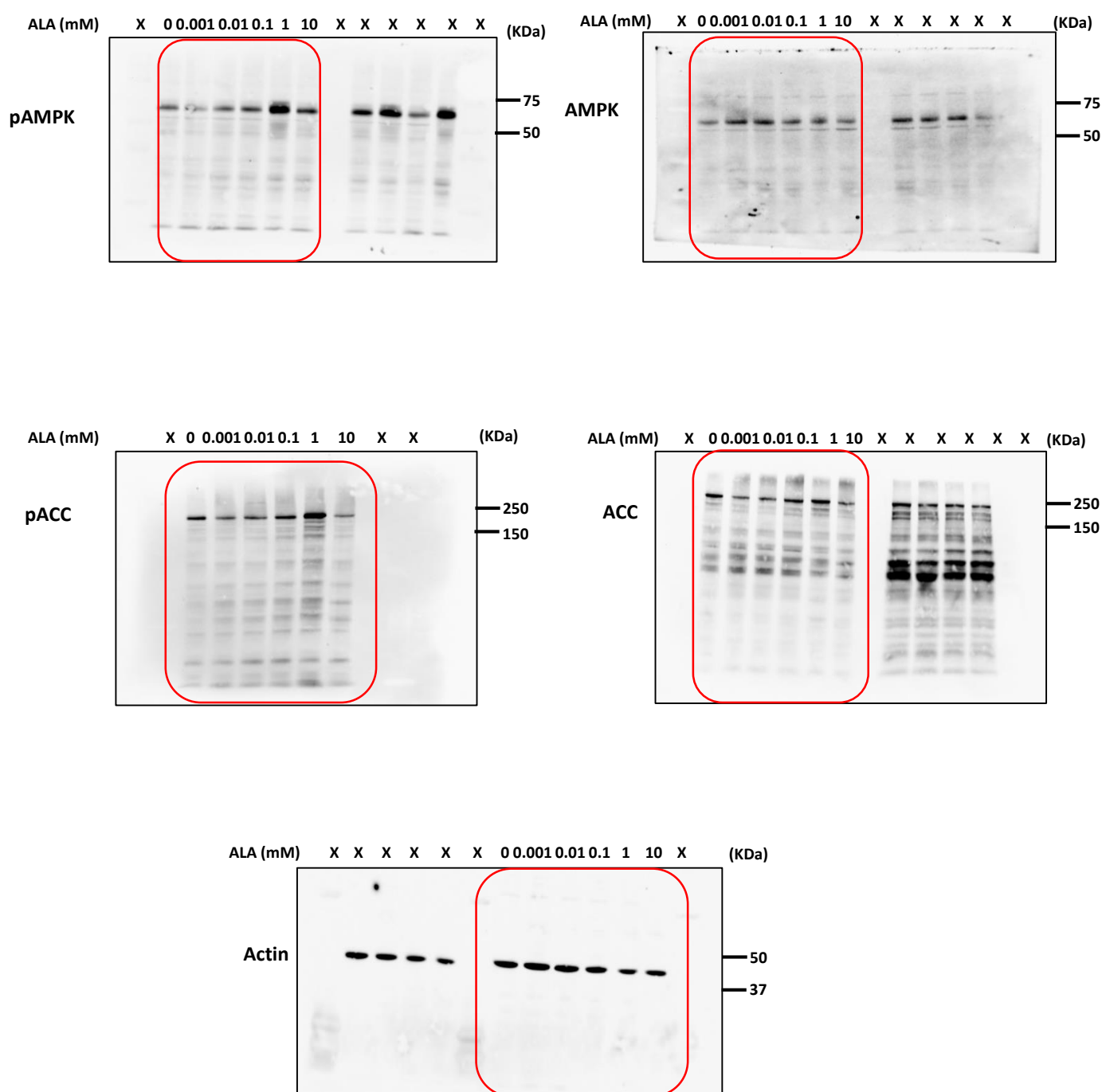

Fig. 4

A

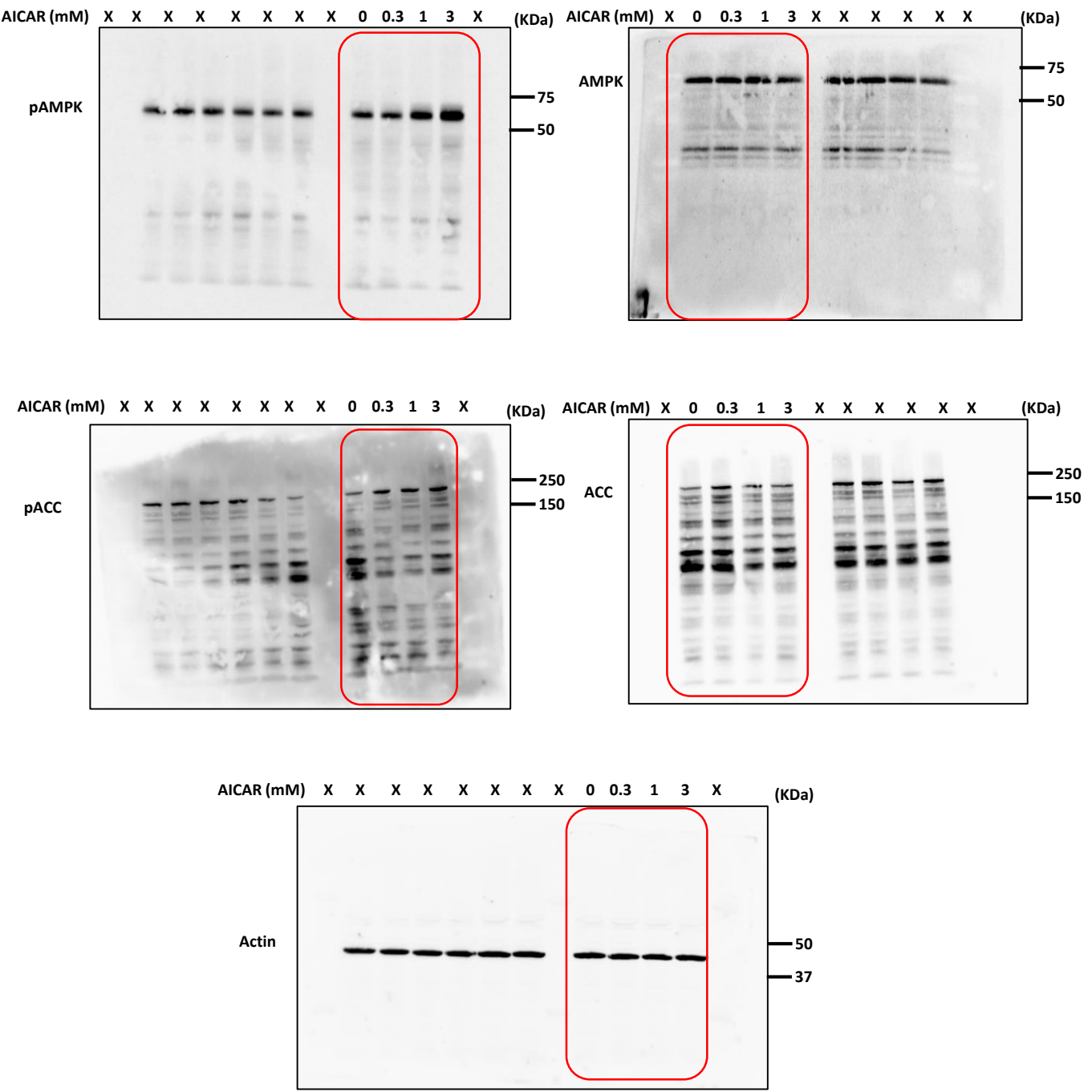

Fig. 5  
A

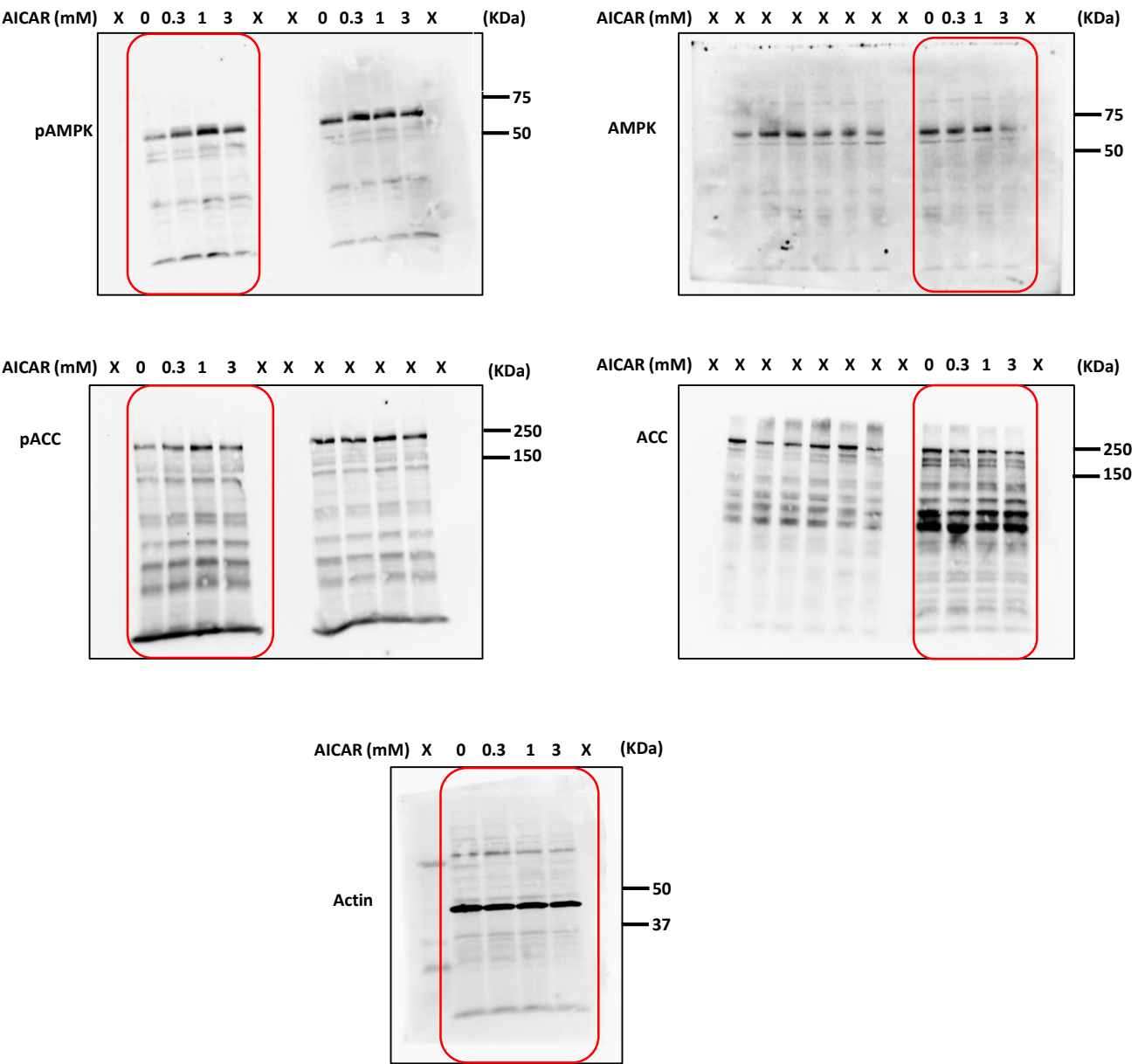

Fig. 6  
A

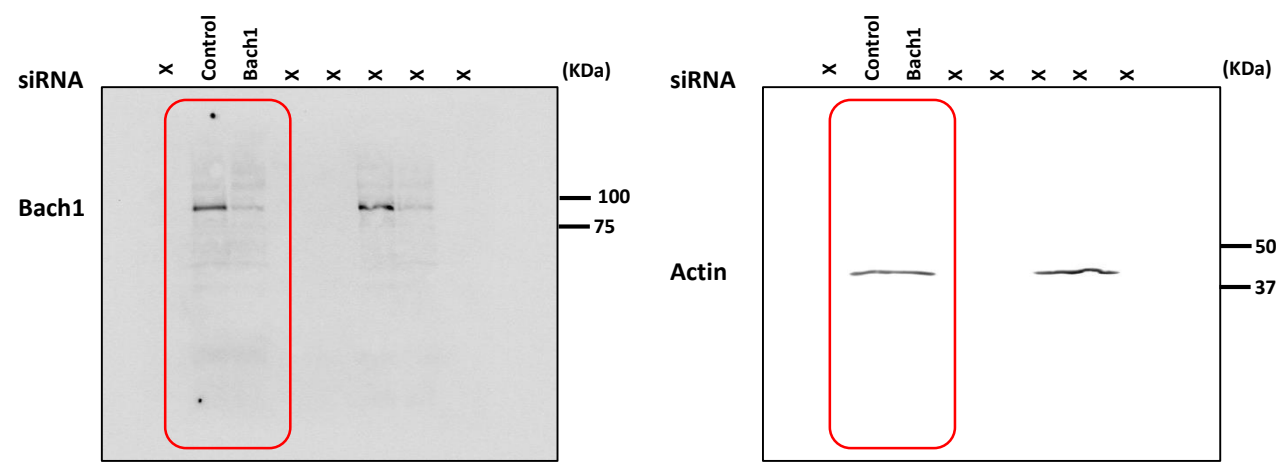

Fig. 7

A

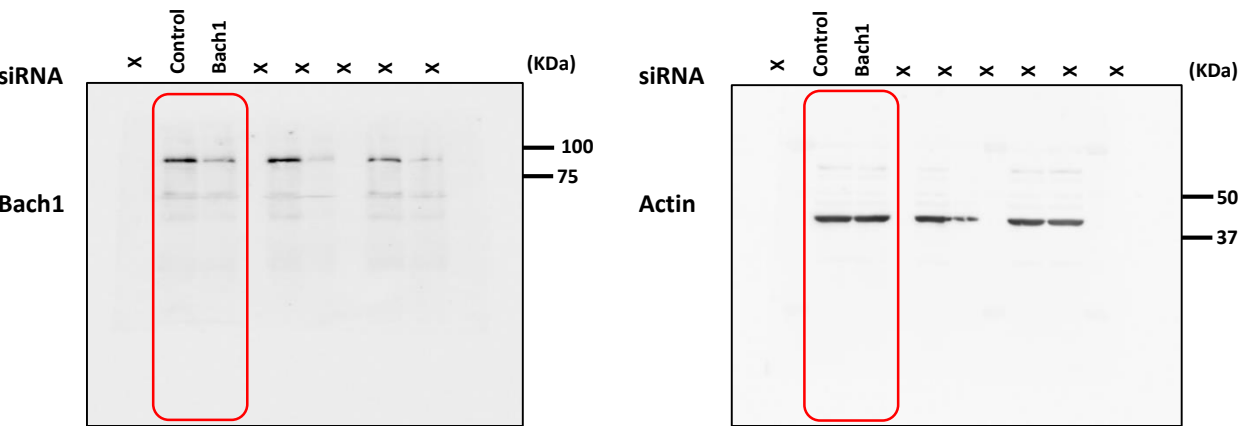

Supplement: S1 File — (PDF) [file pone.0260400.s001.pdf]
